# DBST-FL: Dynamic Behavioural and Semantic Trust for Robust Federated Learning in Industrial IoT

**DOI:** 10.3390/s26154712

**Published:** 2026-07-24

**Authors:** Ammar Alazab, Abin Kumbalapalliyil Tom, Tony Jan, Md Whaiduzzaman, Thien Nguyen, Ansam Khraisat, Ali Almazrouei

**Affiliations:** 1 Centre for Artificial Intelligence Research and Optimization (AIRO), Torrens University Australia (TUA), 46–52 Mountain Street, Ultimo, NSW 2007, Australia; 2 School of Information Technology, Deakin University, Melbourne Burwood Campus, Burwood, VIC 3125, Australia; 3 CyberNex, Somerton, VIC 3062, Australia

**Keywords:** federated learning, Industrial Internet of Things, dynamic behavioural and semantic trust, behavioural trust, semantic trust validation, multiplicative trust fusion, poisoning attack mitigation, backdoor detection, adversarial robustness, IIoT security

## Abstract

Federated learning (FL) has emerged as an effective paradigm for collaborative model training in Industrial Internet of Things (IIoT) environments by enabling distributed devices to learn shared models without exchanging raw data. However, existing FL defence mechanisms predominantly rely on either behavioural analysis of client updates or semantic validation of model performance, limiting their ability to detect sophisticated poisoning and stealthy backdoor attacks that evade single-dimensional trust assessment. This paper proposes DBST-FL, a dynamic behavioural and semantic trust framework for robust federated learning in the Industrial IoT. The proposed framework evaluates each client through two complementary trust dimensions: a behavioural trust layer that measures gradient alignment, historical consistency, and collective deviation and a semantic trust layer that assesses benign utility and template-free semantic stress validation using server-side data. The two trust scores are integrated through a non-compensatory multiplicative trust fusion mechanism, ensuring that weaknesses in one trust dimension cannot be masked by strengths in the other. The resulting trust score guides a trust-aware aggregation strategy that reduces the influence of malicious participants while preserving the contributions of reliable clients. Extensive experiments are conducted on the Edge-IIoTset and UNSW-NB15 datasets using ANN, 1D-CNN, and LSTM models under multiple poisoning and backdoor attack scenarios. The proposed framework achieves overall classification performance competitive with the strongest robust aggregation baselines while consistently delivering stronger resilience against adversarial attacks and lower backdoor attack success rates than representative trust-based and Byzantine-robust aggregation methods, all while maintaining linear per-round computational complexity suitable for large-scale IIoT deployments. The results demonstrate that integrating behavioural and semantic trust within a unified aggregation framework provides an effective and scalable defence against advanced adversarial threats in federated learning.

## 1. Introduction

The Industrial Internet of Things (IIoT) underpins smart manufacturing, critical infrastructure, and industrial automation by linking heterogeneous edge devices through real-time sensing and actuation [1,2]. The operational data generated in these environments are valuable for predictive analytics, yet centralised collection is technically infeasible and legally restricted under prevailing data-governance frameworks [3,4,5,6]. Federated learning (FL) addresses this by enabling devices to collaboratively train shared models, exchanging only model parameters rather than raw data.

The appeal of FL in the IIoT rests, however, on an assumption that is untenable under adversarial conditions: that participating devices behave honestly [7,8,9]. Adversarial clients can pursue poisoning attacks—corrupting training data or manipulating gradient updates—or *backdoor attacks*, which preserve clean-task performance while embedding trigger-conditioned misclassification [10,11,12,13,14]. Stealthy variants specifically mimic the statistical properties of benign contributions, making them the most difficult class to detect.

Three principal lines of defence exist. Statistical and Byzantine-robust aggregation methods (Krum, median, and trimmed mean) provide fault tolerance under IID conditions [15,16]. Trust- and reputation-based approaches extend this with gradient similarity scoring and historical tracking [17,18,19]. Semantic-aware methods [20] attempt functional validation through post-aggregation detection layers. Despite these advances, a fundamental gap remains: *existing defences evaluate trust from either behavioural gradient-space characteristics or semantic functional outcomes but rarely from both simultaneously within the aggregation decision itself*.

Behavioural-only methods such as FLTrust [17] and FedRRA [19] evaluate gradient-space statistics and cannot detect updates that preserve statistical alignment while embedding harmful functionality, the defining characteristic of stealthy backdoor attacks. Semantic-only methods detect functional harm but miss aggressive model poisoning that corrupts gradient statistics. This paper proposes DBST-FL, a dynamic behavioural and semantic trust federated learning framework that addresses both dimensions simultaneously through *multiplicative trust fusion*: behavioural trust $B_{i}^{t}$ (gradient alignment, temporal consistency, and deviation norms) multiplied by semantic trust $S_{i}^{t}$ (functional correctness on clean and trigger-sensitive validation data). Neither dimension can compensate for weakness in the other, a formally established non-compensatory property.

### 1.1. Summary of Contributions

- A **dynamic behavioural and semantic trust aggregation framework** that combines behavioural and semantic trust assessment within the aggregation decision, addressing both gradient-space anomalies and functional semantic harm simultaneously.
- A **multiplicative trust fusion rule** with three formal properties: non-compensation (Proposition 1), boundedness (Proposition 2), and adversarial suppression (Proposition 3), properties that cannot be claimed by additive trust schemes.
- **Trigger-agnostic semantic validation** embedded within the aggregation loop, using validation datasets constructed from synthetic perturbations and domain anomaly injections without requiring knowledge of specific trigger patterns.
- **Linear $O(N(d\;+\;|D_{\mathrm{val}}\left|)\right)$ per-round complexity**, compared with $O(N^{2}d)$ for Krum and Bulyan [21], enabling deployment at the IIoT scale.
- **Comprehensive empirical validation** across 36 experimental conditions (six attacks × three architectures × two datasets) on two public IIoT datasets with full ablation and sensitivity analysis.

### 1.2. Paper Organisation

Section 2 critically reviews the related work. Section 3 presents the DBST-FL framework and theoretical analysis. Section 4 provides security analysis. Section 5 presents the experimental evaluation. Section 6 discusses the limitations. Section 7 concludes the paper.

## 2. Related Work

**Figure 1:**
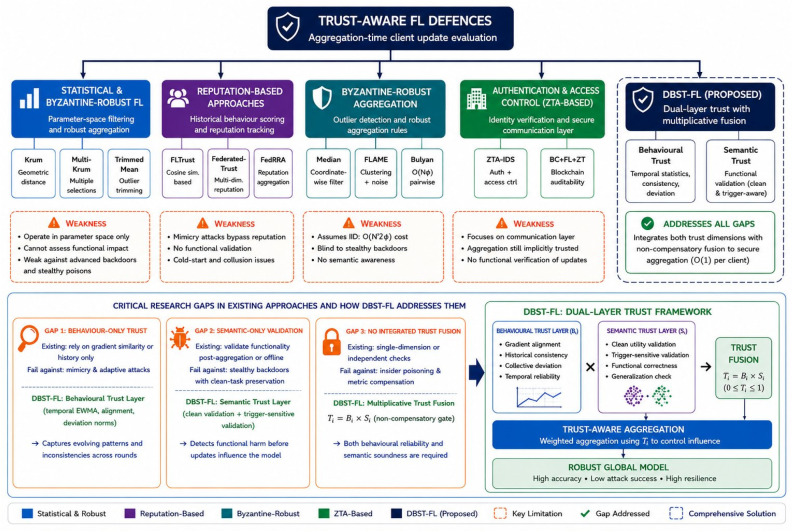
Taxonomy of trust-aware federated learning defences and the three critical research gaps addressed by DBST-FL. **Gap 1**: insufficient trust granularity in statistical methods. **Gap 2**: absence of in-loop semantic validation. **Gap 3**: incomplete zero-trust coverage of the aggregation computation. DBST-FL (right panel) is designed to address all three simultaneously through dynamic behavioural–semantic multiplicative trust fusion.

Figure 1 presents a taxonomy of trust-aware FL defences and the three research gaps that motivated the development of DBST-FL. Table 1 provides a structured comparative reference. This section analyses each defence family critically, emphasising the specific attack vectors each fails against.

### 2.1. Statistical and Byzantine-Robust Aggregation

The coordinate-wise median and trimmed mean methods aggregate parameters using robust statistics, providing tolerance against a bounded adversarial fraction under IID conditions [16]. The Krum and multi-Krum approaches select updates closest to the majority of the neighbours, offering formal Byzantine guarantees when the adversarial fraction is bounded [15]. FLAME [22] extends this family with clustering-based noise injection targeting backdoor attacks. The Bulyan method [21] further strengthens Byzantine-resilient aggregation by combining a Byzantine-resilient selection rule with coordinate-wise filtering, reducing the high-dimensional vulnerability that remains in earlier robust aggregation methods. Because the Bulyan approach requires repeated selection and pairwise distance computation, its computational cost remains $O(N^{2}d)$ for the number of clients, making it less attractive for large-scale IIoT federations.

These methods share a structural limitation: their tolerance proofs require IID data distributions and evaluate updates in gradient space, not in functional output space. Constrain-and-scale attacks reduce the update magnitude to match benign contributions while retaining harmful functionality; projection attacks align the gradient direction while embedding malicious components in perpendicular subspaces. The FLAME method’s noise injection is applied uniformly rather than guided by functional evaluation. The $O(N^{2}d)$ complexity of Krum and Bulyan [21] further limits scalability in large IIoT federations.

### 2.2. Trust-Aware and Reputation-Based Approaches

FLTrust [17] introduced server-side gradient reference alignment, computing cosine similarity between each client’s update and a reference gradient from a small server dataset. FederatedTrust [18] extended this to multi-dimensional reputation tracking. FedRRA [19] distributes reputation through consensus. OptiGradTrust [23] uses reinforcement learning to weight multi-feature gradient-space trust signals adaptively.

Each method evaluates trust exclusively in a gradient parameter space. The key vulnerability is the *mimicry attack*, or an adversary submitting $g_{\mathrm{adv}}=\alpha r+\beta g_{\mathrm{bd}}$, where r is the server reference direction and α≫β achieves high cosine similarity while propagating the $\beta g_{\mathrm{bd}}$ backdoor term. Reputation-based methods are additionally vulnerable to intermittent attacks in which adversaries accumulate trust during benign rounds and spend it during attack rounds.

Recent trust-aware FL approaches have extended reputation scoring beyond simple gradient similarity. FederatedTrust [18] evaluates trustworthiness through multiple FL-specific dimensions, including robustness, fairness, explainability, accountability, privacy, and federation-level indicators. FedSCS [24] introduces stable cosine similarity to improve robustness against unreliable or adversarial client updates. TAIM [25] incorporates client trust into an incentive mechanism for heterogeneous edge clients and uses confidence-based filtering to reduce the impact of unreliable updates; it is primarily an incentive and soft-filtering mechanism rather than a specialised backdoor defence. However, these approaches primarily assess either behavioural reliability, reputation, or contribution credibility, and they do not explicitly integrate trigger-sensitive semantic validation into the aggregation decision. In contrast, DBST-FL combines behavioural and semantic trust through multiplicative fusion, ensuring that gradient-space reliability cannot compensate for functional semantic risk.

### 2.3. Semantic-Aware Federated Learning

Activation clustering and embedding-based analysis can detect updates that alter internal model representations consistently with backdoor behaviour [20,26,27]. Semantic communication integration with FL reduces information overhead [28,29,30]. These approaches face two practical constraints: expensive layer-wise inspection is impractical for real-time IIoT aggregation, and post hoc operation outside the aggregation pipeline means that harmful updates may influence the global model before detection completes. DBST-FL embeds semantic evaluation inside the aggregation decision, preventing any contribution before trust is confirmed.

### 2.4. Authentication-Layer and ZTA-Based Defences

ZTA-based FL frameworks have strengthened identity verification and access control [31,32,33,34,35,36,37,38,39,40]. All existing ZTA-FL work shares a critical structural limitation: ZTA verification applies at the communication boundary only. The aggregation step implicitly trusts all authenticated updates, violating the zero-trust principle—*never trust, always verify*—at the most consequential decision point in FL. DBST-FL extends continuous verification from the communication layer into the aggregation computation itself.

### 2.5. Research Gaps and Positioning

Three structural limitations emerge from the analysis above:

- **Insufficient trust granularity**: Statistical methods cannot distinguish gradient-benign from functionally harmful updates, the exact distinction exploited by stealthy backdoor attacks.
- **Absent in-loop semantic validation**: Semantic-aware methods operate as post hoc layers rather than as components of the aggregation decision.
- **Incomplete zero-trust realisation**: ZTA-FL improves communication security, but aggregation continues under implicit trust.

**Table 1:** Comparative positioning of DBST-FL against representative federated learning defences. Behav. = behavioural trust; ZTA Scope = zero-trust integration extent; BD Detect. = backdoor detection capability. Comparison criteria: (1) *behavioural trust*, whether the method scores gradient-space consistency or historical reliability; (2) *semantic validation*, whether the method assesses functional model correctness on validation or trigger-sensitive data; (3) *ZTA scope*, whether zero-trust principles are applied only at authentication (Auth. only), partially within aggregation (Partial), or at both layers (Full); and (4) *backdoor detection*, empirical ASR reduction capability, rated none, limited, partial, or full based on reported results in cited publications. Entries reflect structural design properties; quantitative comparisons are given in Tables 5 and 6.

Method	Behav.	Semantic	ZTA Scope	BD Detect.	Principal Limitation
FLTrust [17]	Yes	No	Partial	Limited	Trust from cosine similarity. Mimicry attacks achieve high scores while embedding harmful functionality.
FederatedTrust [18]	Yes	No	Partial	Limited	Intermittent adversaries accumulate trust during benign rounds; no functional evaluation.
FedRRA [19]	Yes	No	Partial	Limited	Distributed reputation consensus. Coordinated clients manipulate scoring; stealthy backdoors accumulate positive reputations.
OptiGradTrust [23]	Yes	No	Partial	Partial	RL-based gradient-space trust. Cannot detect functionally harmful but statistically aligned updates.
Semantic-aware FL [26,27]	No	Yes	None	Partial	Post hoc activation clustering. Harmful updates may influence global model before detection; unsuitable for real-time aggregation.
FLAME [22]	Yes	No	None	Partial	Constrain-and-scale attacks reduce magnitude to pass filter while retaining backdoor functionality.
ZTA-based FL [31,32]	No	No	Auth. only	None	Verification restricted to the authentication layer. No behavioural or semantic trust evaluation at aggregation.
**DBST-FL (this work)**	**Yes (temporal)**	**Yes (template-free)**	**Full ^†^**	**Yes**	Requires server-side validation data. Detection may delay for novel triggers; coalition influence bounded but not fully eliminated.

Note: **Bold** row indicates the proposed method (DBST-FL, this work). Yes, Partial, and No entries based on the primary mechanism described in cited publications. ^†^ Full refers to zero-trust-inspired verification at both the communication and aggregation stages; it does not imply a complete enterprise-grade ZTA implementation.

Table 1 confirms that no existing method simultaneously provides behavioural trust evaluation, in-loop semantic validation, zero-trust-inspired aggregation verification, and reliable backdoor detection. DBST-FL is designed to close all three structural gaps within a linear-complexity per-round framework.

## 3. The DBST-FL Framework

### 3.1. System and Operational Assumptions

We consider *N* distributed IIoT clients, each holding a private local dataset $D_{i}$ drawn from a distribution $P(x,y|c_{i})$. Data distributions are non-IID across clients. The global training objective minimises the aggregate empirical loss:

(1) $$\theta^{*}=\arg\min_{\theta }\;F\left(\theta \right)=\sum_{i=1}^{N}\frac{|D_{i}|}{\left|D\right|}\;F_{i}\left(\theta \right),\;F_{i}\left(\theta \right)=E_{\left(x,y\right)∼D_{i}}\left[\ell \left(\theta ;\;x,y\right)\right]$$

where θ denotes the global model parameters, *N* is the number of clients, $D_{i}$ is the local dataset of client *i*, $D={⋃}_{i}D_{i}$ is the union of all local data, $F_{i}\left(\theta \right)$ is the local empirical loss, and ℓ(θ;x,y) is the per-sample loss (e.g., cross-entropy). The weighting $|D_{i}\left|/|D\right|$ ensures that clients with more data contribute proportionally to the global optimum.

At each round *t*, the server broadcasts $\theta_{\mathrm{global}}^{t}$, and clients compute local updates $\theta_{i}^{t}$ and transmit them for aggregation. The server is honest but curious. Standard ZTA controls (mutual authentication, freshness validation, and integrity checks [32]) are enforced at the communication boundary and are necessary but insufficient against poisoning or backdoor attacks from authenticated clients.

Adversaries control up to 30% of clients and may coordinate strategies. Three attack modes are considered: data poisoning (up to 20% of local samples corrupted); model poisoning (up to 10% of update entries manipulated); and backdoor insertion (5% of parameters modified, trigger injected into 1–2% of local samples).

### 3.2. Architecture Overview

**Figure 2:**
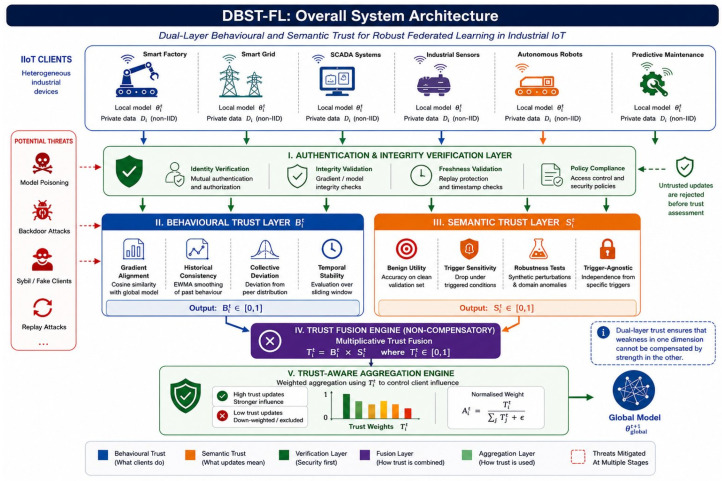
DBST-FL’soverall system architecture, showing five processing layers. **Layer I**: Authentication and integrity verification rejects untrusted updates before trust assessment. **Layer II**: Behavioural trust layer $B_{i}^{t}$ evaluates gradient alignment, historical consistency, collective deviation, and temporal stability. **Layer III**: Semantic trust layer $S_{i}^{t}$ evaluates benign utility, trigger sensitivity, robustness, and trigger-agnostic correctness. **Layer IV**: Trust fusion engine applies non-compensatory multiplicative fusion $T_{i}^{t}=B_{i}^{t}\times S_{i}^{t}\in \left[0,1\right]$. **Layer V**: Trust-aware aggregation engine computes normalised weights $A_{i}^{t}$ and produces the robust global model $\theta_{\mathrm{global}}^{t+1}$.

Figure 2 presents the complete DBST-FL architecture. All client updates traverse a zero-trust authentication gate before dynamic behavioural and semantic trust evaluation. Behavioural and semantic trust scores are computed for each client and combined multiplicatively. The global model is updated only after all trust evaluations for the current round are finalised.

### 3.3. Theoretical Justification: Multiplicative Trust Fusion

Let the trust scores be bounded:

(2) $$0\leq B_{i}^{t}\leq 1,\;0\leq S_{i}^{t}\leq 1$$

Here, $B_{i}^{t}\in \left[0,1\right]$ measures the gradient-space statistical reliability, and $S_{i}^{t}\in \left[0,1\right]$ measures functional semantic correctness; both are computed independently before aggregation.

The joint trust score is defined as follows:

(3) $$T_{i}^{t}=B_{i}^{t}\times S_{i}^{t}$$

The product structure enforces that $T_{i}^{t}$ is high only when both $B_{i}^{t}$ and $S_{i}^{t}$ are simultaneously high; a near-zero value for either factor drives the joint score toward zero regardless of the other’s value (Proposition 1).

**Figure 3:**
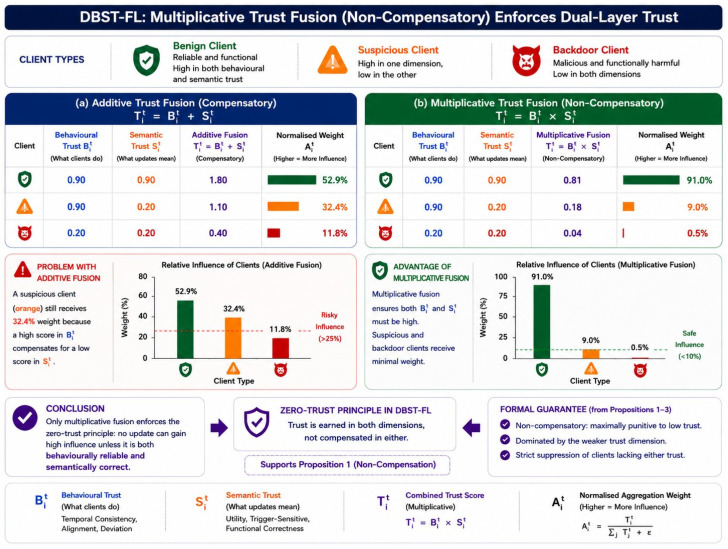
Comparison of additive and multiplicative trust fusion for three client types: benign (B=0.90, S=0.90), suspicious (B=0.90, S=0.20), and malicious backdoor (B=0.20, S=0.20). Under additive fusion (**left**), the suspicious client receives 32.4% of the normalised aggregation weight because a high $B_{i}^{t}$ vlaue compensates for low $S_{i}^{t}$ values. Under multiplicative fusion (**right**, DBST-FL), the same client receives only 9.0% of the weight, and the malicious client receives 0.5%. Propositions 1–3 formally establish that neither trust dimension can compensate for weakness in the other.

The additive alternative with a weighting parameter λ∈[0,1] yields $T_{i}^{t}=\lambda B_{i}^{t}+\left(1-\lambda \right)S_{i}^{t}$. This formulation is *compensatory*: when $B_{i}^{t}=1$, $S_{i}^{t}=0$, and λ=0.5, the additive score yields 0.5, while the multiplicative score yields zero. Figure 3 illustrates this consequence across three representative client scenarios.

Three formal properties formally capture the non-compensatory gating behaviour of multiplicative fusion for adversarial FL.

**Proposition** **1** (Non-compensation)**.** *For any client i at round t, if either $B_{i}^{t}$ or $S_{i}^{t}$ approaches zero, the joint trust score also approaches zero: $\lim_{B_{i}^{t}\to 0}T_{i}^{t}=0$ and $\lim_{S_{i}^{t}\to 0}T_{i}^{t}=0$.*

**Proof.**  Since $T_{i}^{t}=B_{i}^{t}\times S_{i}^{t}$, the vanishing of either factor forces the product to zero. High behavioural trust cannot compensate for low semantic trust, and vice versa. This property cannot be satisfied by any additive scheme for λ∈(0,1). □

Proposition 1 is the key differentiator; a stealthy backdoor client with high gradient alignment ($B_{i}^{t}\approx 1$) but low functional correctness ($S_{i}^{t}\approx 0$) receives near-zero joint trust, regardless of its gradient-space properties.

**Proposition** **2** (Boundedness and stability)**.** *If Equation *(2)* holds, then* $T_{i}^{t}$ *satisfies* $0\leq T_{i}^{t}\leq 1$*, and the resulting normalised aggregation weights remain numerically stable across training rounds.*

**Proof.**  The product of two values in [0,1] lies in [0,1]. The normalised weight $A_{i}^{t}=T_{i}^{t}/\sum_{j=1}^{N}T_{j}^{t}$ is well-defined and stable, provided at least one client has non-zero trust. □

**Proposition** **3** (Adversarial suppression)**.** *Suppose $S_{i}^{t}\leq \varepsilon$ for some ε>0. Since $B_{i}^{t}\leq 1$, the joint trust score satisfies $T_{i}^{t}=B_{i}^{t}\times S_{i}^{t}\leq \varepsilon$, regardless of $B_{i}^{t}$.*

**Proof.**  The proof is direct from the multiplicative definition and the bound $B_{i}^{t}\leq 1$. □

### 3.4. Behavioural Trust Estimation

**Figure 4:**
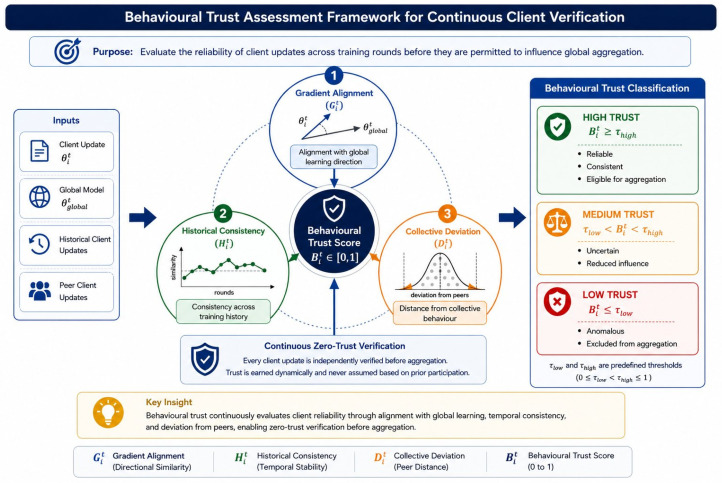
Behavioural trust assessment framework. Three independent indicators—gradient alignment ${\bar{G}}_{i}^{t}$, historical consistency $H_{i}^{t}$ (EWMA-smoothed), and collective deviation $D_{i}^{t}$ (Gaussian-penalised)—are combined by weighted sum to produce $B_{i}^{t}\in \left[0,1\right]$. Three tiers result from this: high trust ($B_{i}^{t}\geq \tau_{\mathrm{high}}$, full aggregation), medium trust ($\tau_{\mathrm{low}}<B_{i}^{t}<\tau_{\mathrm{high}}$, reduced influence), and low trust ($B_{i}^{t}\leq \tau_{\mathrm{low}}$, excluded).

Behavioural trust $B_{i}^{t}$ quantifies the statistical reliability of each client’s update through three complementary indicators, as illustrated in Figure 4.

#### 3.4.1. Gradient Alignment

(4) $${\bar{G}}_{i}^{t}=\max\;\left(0,\;\frac{\Delta w_{i}^{t}\cdot \Delta w_{\mathrm{ref}}^{t}}{∥\Delta w_{i}^{t}∥\;∥\Delta w_{\mathrm{ref}}^{t}∥}\right)$$

where $\Delta w_{i}^{t}=\theta_{i}^{t}-\theta_{\mathrm{global}}^{t-1}$ is the parameter update vector for client *i* and $\Delta w_{\mathrm{ref}}^{t}=\frac{1}{|C_{\mathrm{ref}}|}\sum_{j\in C_{\mathrm{ref}}}\Delta w_{j}^{t}$ is a reference direction computed as the coordinate-wise *trimmed mean* of updates from a randomly sampled reference subset $C_{\mathrm{ref}}$ of max(10,⌈0.3N⌉) clients at each round (giving at least 10 clients with N=30), with a trimming ratio ρ=0.1 applied coordinate-wise. Using a minimum pool of 10 clients ensures stable estimation under the 30% adversarial threat model; clients whose trust score falls below τ in the previous round are excluded from $C_{\mathrm{ref}}$ in subsequent rounds, progressively purifying the reference pool. Using update vectors rather than raw parameters avoids the trivial alignment that arises from comparing full parameter vectors. The max(0,·) clamp ensures that ${\bar{G}}_{i}^{t}\in \left[0,1\right]$.

#### 3.4.2. Historical Consistency (EWMA)

(5) $$H_{i}^{t}=\gamma \;H_{i,\mathrm{curr}}^{t}+\left(1-\gamma \right)\;H_{i}^{t-1},\;\gamma \in \left[0,1\right]$$

where $H_{i,\mathrm{curr}}^{t}=\cos\left(\Delta w_{i}^{t},\bar{\Delta }w^{t}\right)$ is the raw cosine similarity between client *i*’s update and the round-average update, $H_{i}^{t-1}$ is the smoothed value from the previous round initialised to 0.5, and γ∈[0,1] controls the trade-off between responsiveness and stability. The sensitivity analysis confirms stable performance for γ∈[0.6,0.8], with γ=0.7 as the empirically optimal setting.

#### 3.4.3. Collective Deviation

(6) $$D_{i}^{t}=\exp\;\left(-\frac{∥\Delta w_{i}^{t}-\mu^{t}{∥}^{2}}{2\sigma^{2}}\right)$$

where $\mu^{t}=\frac{1}{N}\sum_{i}\Delta w_{i}^{t}$ is the mean update across all clients and $\sigma^{2}$ is the empirical variance of update norms in round *t*, i.e., $\sigma^{2}=\frac{1}{N}\sum_{i}{∥\Delta w_{i}^{t}-\mu^{t}∥}^{2}$, estimated fresh each round. The Gaussian penalisation smoothly suppresses large deviations without a hard rejection threshold, which is important under non-IID data.

#### 3.4.4. Behavioural Trust Score

(7) $$B_{i}^{t}=w_{1}{\bar{G}}_{i}^{t}+w_{2}H_{i}^{t}+w_{3}D_{i}^{t},\;w_{1}+w_{2}+w_{3}=1$$

where $w_{1},w_{2},w_{3}\geq 0$ are non-negative weights summing to unity, controlling the relative contribution of each indicator; equal weighting ($w_{1}=w_{2}=w_{3}=1/3$) is used as the default. The role of Equation (7) is to compress three heterogeneous indicators—each in [0,1]—into a single scalar $B_{i}^{t}\in \left[0,1\right]$ that represents the client’s overall gradient-space reliability. A high $B_{i}^{t}$ value (close to one) indicates that the client’s update is well aligned with the global direction, historically consistent, and not an outlier relative to peers. A low $B_{i}^{t}$ value (close to zero) indicates at least one dimension of anomalous behaviour, which under multiplicative fusion (Equation (3)) directly suppresses that client’s aggregation weight. Sensitivity analysis confirmed robustness to moderate weight variations of ±20%.

Clients with $B_{i}^{t}<\tau_{\mathrm{low}}=0.6$ are excluded from the current round’s aggregation. The clients in [0.6,0.8) are down-weighted proportionally. The sensitivity analysis shows stable detection performance for τ∈[0.75,0.85], justifying the default τ=0.8.

### 3.5. Semantic Trust Computation

**Figure 5:**
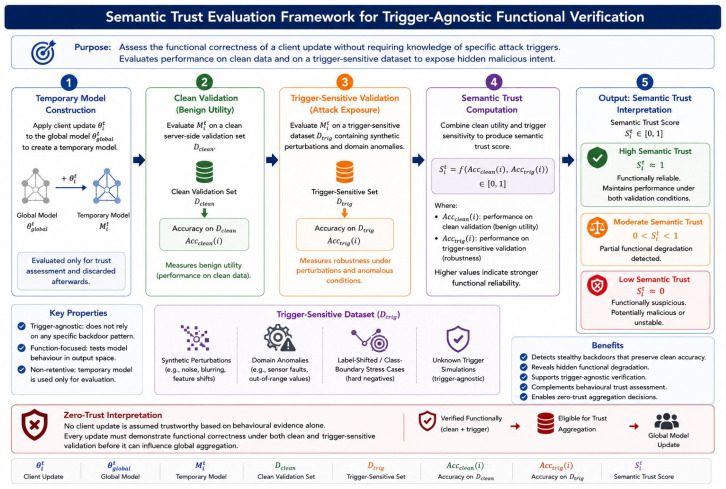
Semantic trust evaluation framework for trigger-agnostic functional verification. A temporary model $M_{i}^{t}$ is constructed by applying $\theta_{i}^{t}$ to the global model (used only for scoring and discarded afterwards). The benign utility ${\mathrm{Acc}}_{\mathrm{clean}}$ is measured on a clean validation set $D_{\mathrm{val}}$; adversarial sensitivity ${\mathrm{Acc}}_{\mathrm{trig}}$ is measured on a trigger-sensitive set $D_{\mathrm{trig}}$ (synthetic perturbations, domain anomalies, and label-shifted stress cases), with no specific trigger templates required. Semantic trust $S_{i}^{t}=\beta_{1}U_{i}^{t}+\beta_{2}P_{i}^{t}$ is computed from both measurements. $P_{i}^{t}$ measures excess trigger-set accuracy relative to the global model baseline; only deviations above this baseline reduce $P_{i}^{t}$. The global model is not modified until trust-weighted aggregation is complete.

Semantic trust $S_{i}^{t}$ evaluates the functional correctness of each candidate update before it is admitted to aggregation. The trigger-agnostic evaluation workflow is illustrated in Figure 5.

#### 3.5.1. Benign Utility

(8) $$U_{i}^{t}=\mathrm{Acc}\left(\theta_{i}^{t},\;D_{\mathrm{val}}\right)$$

where $\mathrm{Acc}(\theta_{i}^{t},D_{\mathrm{val}})$ denotes the classification accuracy of the global model updated with $\theta_{i}^{t}$ when evaluated on the clean server-side validation set $D_{\mathrm{val}}$. A high $U_{i}^{t}$ value indicates that the update preserves or improves task utility on representative benign inputs.

#### 3.5.2. Adversarial Robustness Score

$D_{\mathrm{trig}}$ is a server-side evaluation set constructed without knowledge of specific attack triggers. It comprises (1) Gaussian-perturbed copies of validation samples ($\sigma_{\mathrm{noise}}=0.1$), (2) domain-anomaly injections using out-of-distribution feature values, and (3) label-shifted stress cases. A backdoored model responds abnormally to these inputs —producing higher confidence or accuracy on them—relative to a clean model [26]. To calibrate the score, $\mathrm{Acc}(\cdot ,D_{\mathrm{trig}})$ is compared against the server’s clean reference model on the same set; only deviations *above* the clean-model baseline are penalised, and thus a genuinely robust model is not penalised for low raw accuracy on difficult samples. The score is defined as follows:

(9) $$P_{i}^{t}=1-\max\;\left(0,\;\mathrm{Acc}\left(\theta_{i}^{t},D_{\mathrm{trig}}\right)-\mathrm{Acc}\left(\theta_{\mathrm{ref}}^{t},D_{\mathrm{trig}}\right)\right)$$

where $\theta_{\mathrm{ref}}^{t}$ is the current global model (serving as the clean reference) and the max(0,·) clamp ensures that a client whose trigger-set accuracy is below the reference receives $P_{i}^{t}=1$ (maximum robustness score) rather than being penalised for correctly rejecting adversarial inputs. Only *excess* trigger-set accuracy above the reference baseline is penalised; a backdoored client activates abnormally on $D_{\mathrm{trig}}$ relative to the global model, producing a high excess and low $P_{i}^{t}$, which reduces $S_{i}^{t}$. A genuinely robust or low-accuracy model that does not exceed the reference is not penalised. The evaluation does not require exact trigger templates; it is designed to detect semantic deviations consistent with backdoor behaviour, specifically excess target-class activation relative to the clean reference. However, this assumption may not hold for all attack families; targeted clean-label backdoors, fully distributed triggers, or stealthy attacks whose activations on synthetic perturbations closely mirror those of the clean reference may evade detection. These scenarios are acknowledged as a limitation (Section 6). Empirical evaluation across the trigger families tested here is provided in Section 5.3.

#### 3.5.3. Semantic Trust Score

(10) $$S_{i}^{t}=\beta_{1}U_{i}^{t}+\beta_{2}P_{i}^{t},\;\beta_{1}+\beta_{2}=1$$

where $\beta_{1},\beta_{2}\geq 0$ with $\beta_{1}+\beta_{2}=1$ weight benign utility $U_{i}^{t}$ against adversarial robustness $P_{i}^{t}$. Since $P_{i}^{t}$ penalises only excess trigger-set activation relative to the reference model, a lower $P_{i}^{t}$ value indicates abnormal trigger-sensitive behaviour, while a higher $P_{i}^{t}$ value indicates semantic consistency with the reference model. Equal weighting ($\beta_{1}=\beta_{2}=0.5$) is the default. $S_{i}^{t}=1$ denotes a fully trusted semantic profile; $S_{i}^{t}\approx 0$ signals a functionally suspicious update.

After evaluation, the temporary model is discarded; only the scalar $S_{i}^{t}$ is retained. The global model is unmodified until trust-weighted aggregation (Section 3.6) is complete.

### 3.6. Trust-Weighted Aggregation


(11) $$\begin{array}{cc} T_{i}^{t} & =B_{i}^{t}\times S_{i}^{t} \end{array}$$

(12) $$\begin{array}{cc} A_{i}^{t} & =\frac{T_{i}^{t}}{\sum_{j=1}^{N}T_{j}^{t}} \end{array}$$

(13) $$\begin{array}{cc} \theta_{\mathrm{global}}^{t+1} & =\sum_{i=1}^{N}A_{i}^{t}\;\theta_{i}^{t} \end{array}$$

Equation (11) computes the joint trust score as the multiplicative product of both layers. Equation (12) normalises all client scores into a probability simplex ($A_{i}^{t}\geq 0$, $\sum_{i}A_{i}^{t}=1$). Therefore, higher-trust clients receive proportionally greater aggregation weight. Equation (13) produces the next global model $\theta_{\mathrm{global}}^{t+1}$ as a trust-weighted average; clients with $B_{i}^{t}$ values below the isolation threshold are assigned $A_{i}^{t}=0$ and excluded entirely.

### 3.7. Convergence Analysis

We provide a convergence analysis under simplified assumptions to offer limited theoretical intuition for the trust-weighted aggregation scheme. **This result should be interpreted as motivation rather than a guarantee**: the strongly-convex Assumptions (A1–A2) do not hold for the ANN, 1D-CNN, and LSTM architectures used in the experiments. A complete non-convex convergence analysis under bounded adversarial perturbation is an open problem and is deferred to future work.

**Proposition** **4** (Convergence under Simplified Assumptions)**.** (Simplified theoretical analysis; the experiments used non-convex ANN, CNN, and LSTM models to which the following convex guarantee does not directly apply). Assume that (A1) each local loss $F_{i}\left(\theta \right)$ is L-smooth; (A2) each $F_{i}$ is strongly convex on μ; (A3) local stochastic gradients are unbiased with bounded variance $\sigma^{2}$; and (A4) the trust-weighted weight $A_{i}^{t}\geq \delta >0$ for all benign clients i (benign clients retain positive influence, a condition that may be weakened under severe non-IID data). Under these conditions, with a decaying learning rate $\eta_{t}=O\left(1/t\right)$, the sequence $\left\{\theta_{\mathrm{global}}^{t}\right\}$ produced by DBST-FL converges to a neighbourhood of the true optimum $\theta^{*}$:

(14) $$E\;\left[F\left(\theta_{\mathrm{global}}^{t}\right)-F\left(\theta^{*}\right)\right]\;\leq \;\frac{C}{t}+\frac{\sigma^{2}}{N_{\mathrm{clean}}\;\mu }$$

*where C>0 is a constant depending on initialisation and L/μ and $N_{\mathrm{clean}}$ is the number of benign clients whose trust scores exceed the isolation threshold τ.*

**Proof.**  Trust-weighted aggregation can be cast as a weighted FedAvg step with time-varying weights $A_{i}^{t}$. Under Assumption (A4), the effective aggregate gradient at each round is a convex combination of benign client gradients with bounded coefficients. Applying the standard convergence analysis for weighted distributed gradient descent [16], which establishes geometric contraction under *L*-smooth strongly convex objectives for mixtures of benign gradients, gives the O(1/t) rate in Equation (14). The additive variance term arises from non-IID heterogeneity across the $N_{\mathrm{clean}}$ retained clients; it vanishes as $N_{\mathrm{clean}}\to \infty$ or when the data are IID. Byzantine clients are suppressed toward the zero weight by Proposition 3, and thus they contribute only a bounded perturbation to the effective gradient that does not affect the asymptotic rate. A full derivation following the framework of [15] is deferred to future work. □

**Remark** **1.** *Proposition 4 provides theoretical grounding for trust-weighted aggregation under idealised convex objectives. Empirically, DBST-FL converges stably within T=100 rounds on both datasets across all non-convex architectures evaluated (ANN, 1D-CNN, and LSTM); we attribute this to the trust mechanism progressively suppressing adversarial gradients (Proposition 3), which reduces effective gradient noise. A convergence analysis for non-convex objectives with adversarial clients is deferred to future work.*

### 3.8. Computational Complexity

Per-round server-side complexity:

(15) $$O\left(Nd\right)+O(N|D_{\mathrm{val}}\left|\right)=O\;\left(N(d+|D_{\mathrm{val}}|)\right)$$

where *d* is the model parameter dimensionality and $|D_{\mathrm{val}}|$ is the cardinality of the server-side validation set. This is linear in the number of clients *N*, contrasting with $O(N^{2}d)$ for Krum and Bulyan [21]. Table 2 compares the complexity across methods.

When $d\;\gg \;|D_{\mathrm{val}}|$ (deep models with a small validation set), DBST-FL’s complexity reduces to the same O(Nd) value as FLTrust and Coordinate-Wise Median. The semantic term scales linearly with *N*, unlike for pairwise methods.

## 4. Security Analysis

### 4.1. Suppression Under Persistent Poisoning

Under persistent poisoning, adversarial updates diverge from the collective gradient direction over multiple rounds, causing ${\bar{G}}_{i}^{t}$ and $D_{i}^{t}$ to decline through Equations (4) and (6). The EWMA component $H_{i}^{t}$ accumulates evidence of a persistent anomaly while suppressing transient non-IID fluctuations.

**Property** **1** (Progressive trust reduction)**.** *If a client repeatedly reduces either $B_{i}^{t}$ or $S_{i}^{t}$ across a trust window of K rounds, then its normalised weight $A_{i}^{t}$ decreases monotonically relative to clients with stable trust. This follows from the monotonicity of the multiplicative trust score and the normalisation in Equation* (12)*.*

Experimental evidence (Section 5.2): DBST-FL maintains a mean accuracy of 0.9475 on Edge-IIoTset under data and model poisoning compared with 0.8472 for Adaptive FedAvg, a gap of 7.6 percentage points attributable to DBST-FL’s accumulation of behavioural evidence.

### 4.2. Suppression of Stealthy Backdoor Attacks

Backdoor attacks preserve gradient alignment (high $B_{i}^{t}$) while embedding trigger-conditioned misclassification. The semantic trust $P_{i}^{t}$ in Equation (9) detects this configuration without requiring trigger templates. Under Proposition 3, we also have the following:

**Property** **2** (Semantic suppression)**.** *A backdoor client with $S_{i}^{t}\leq \varepsilon$ receives $T_{i}^{t}\leq \varepsilon$ regardless of $B_{i}^{t}$. This suppression is not achievable by any additive scheme when ε≪λ.*

Experimental evidence (Section 5.3): DBST-FL achieved an ASR = 2.00% under a trigger-column backdoor versus 15.0% for FederatedTrust [18] and 10.0% for TAIM [25]. The gap to FederatedTrust directly quantifies the marginal contribution of the semantic trust layer.

### 4.3. Colluding Adversaries: Bounded but Not Eliminated

When adversaries coordinate strategies, protection is bounded rather than absolute. Stress tests (Section 5.5) show coordinated adversaries retaining 45.7–48.6% of the total aggregation weight despite DBST-FL maintaining an accuracy of 0.9443–0.9798. A highly coordinated coalition that simultaneously preserves gradient alignment and functional plausibility represents the general information-theoretic limit of aggregation-based defences [13].

## 5. Experimental Evaluation

### 5.1. Experimental Set-Up

#### 5.1.1. Implementation and Environment

DBST-FL was implemented in Python 3.11 using PyTorch 2.1. FL training was simulated on a 64-bit workstation (Intel Core i5, 32 GB of RAM). Each experiment ran for T=100 communication rounds with E=5 local epochs per round and a mini-batch size B=64. The Adam optimiser was used with a learning rate $\eta =10^{-3}$ and default momentum parameters. Data were split into 70% training, 10% validation, and 20% test per client; the server-side validation set $D_{\mathrm{val}}$ comprised 5% of the total samples drawn uniformly from the global distribution, and $D_{\mathrm{trig}}$ contained 500 synthetic trigger- sensitive samples constructed as described in Section 3. In practice, $D_{\mathrm{val}}$ can be obtained from public IIoT calibration data, consented validation traces, or synthetic data generated from publicly available domain specifications, without requiring access to any client’s private data. The trust window K=5, and the isolation threshold τ=0.8. The results represent the means over five independent runs with fixed random seeds (1, 2, 3, 4, and 5); the per-method standard deviations of the accuracy metric are reported alongside the mean values in Table 5 (range: 0.0019–0.0079 across all evaluated methods and both datasets).

#### 5.1.2. Datasets

*Edge-IIoTset* [41] is a feature-rich IIoT telemetry dataset containing 15 attack categories. *UNSW-NB15* [42] is a heterogeneous network traffic dataset with 49 features and 9 attack types. Both datasets were partitioned using non-IID Dirichlet splits (concentration α=0.5) across 30 virtual clients.

#### 5.1.3. Model Architectures

Three architectures reflect the computational range of IIoT devices: ANN (lightweight tabular baseline), 1D-CNN (local sequential patterns), and LSTM (temporal dependencies). Table 3 provides the full architecture details.

#### 5.1.4. Attack Configurations and Baselines

Six adversarial settings were evaluated: label flipping, clean-label poisoning, adaptive model poisoning, explicit model overwriting, trigger-column backdoor, and semantic backdoor. All experiments used 30 clients with nine adversarial participants (30%). Baselines included FedAvg [43], Weighted FedAvg, Adaptive FedAvg, Krum [15], Multi-Krum [15], Coordinate-Wise Median [16], Trimmed Mean [16], FLAME [22], FederatedTrust [18], TAIM [25] (a trust-aware incentive and soft-filtering mechanism, included as a trust-reliability baseline rather than a specialised backdoor defence), BC + FL + Trust [44] (our shorthand for the blockchain-enabled federated trust framework of [44]), and FedSCS [24].

The evaluated baselines cover four defence families: (1) *standard averaging*: FedAvg, Weighted FedAvg, and Adaptive FedAvg; (2) *Byzantine-resilient aggregation*: Krum, Multi-Krum, Coordinate-Wise Median, Trimmed Mean, and FLAME; (Bulyan [21] is discussed in the complexity comparison but was not separately evaluated due to its $O(N^{2}d)$ cost and absence from standard IIoT FL benchmarks.) (3) *Trust- or reputation-based*: FLTrust, FederatedTrust, FedSCS, TAIM, and OptiGradTrust; and (4) *blockchain- or ZTA-oriented*: BC + FL + Trust and recent ZTA-based FL approaches. For methods not originally designed for backdoor defence—such as TAIM and BC + FL + Trust—the comparison should be interpreted as a trust-reliability baseline rather than as a specialised backdoor-defence comparison.

Table 4 summarises the implementation status and hyperparameters for all baselines.

#### 5.1.5. Sensitivity Analysis

Trust thresholds τ∈[0.70,0.90] showed stable performance, with a consistent operating region [0.75,0.85]. For the window K∈{3,5,7,9}, K=3 saw faster detection with slightly elevated false positives; K=5 provided the best balance; and K=7–9 reduced false positives at the cost of detection latency. The EWMA factor γ∈[0.6,0.8] maintained robust performance. These results confirm that DBST-FL does not require fine-grained hyperparameter tuning.

### 5.2. Aggregation Robustness

Table 5 reports mean the accuracy, F1 score, and robustness index for all evaluated methods on both datasets under six adversarial attacks using the ANN architecture.

On Edge-IIoTset, DBST-FL achieved the highest mean accuracy (0.9475), F1 score (0.9505), and robustness index (0.9181). Trimmed Mean (0.9212) and Coordinate-Wise Median (0.9183) were the closest conventional competitors, confirming that robust coordinate-wise aggregation provided meaningful but insufficient protection against the full attack mix. The substantially lower performance of Adaptive FedAvg (0.8472) reflects sensitivity to gradient manipulation attacks.

**Figure 6:**
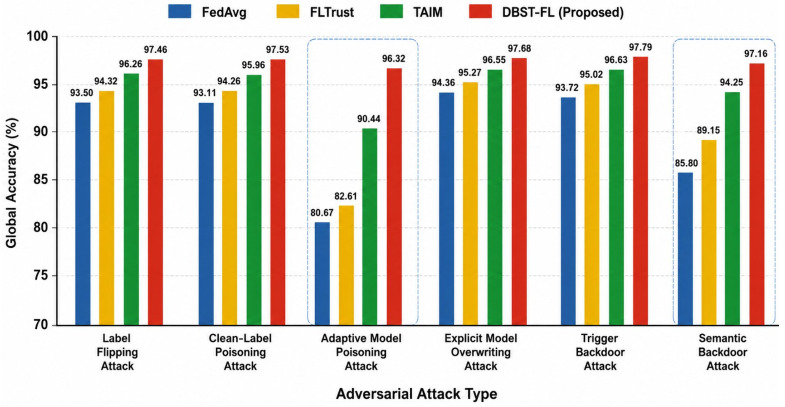
Global accuracy comparison across six adversarial attack types (FedAvg, FLTrust, TAIM, and DBST-FL). Results are averaged over ANN, 1D-CNN, and LSTM architectures on both datasets (30 clients, 9 adversaries). DBST-FL achieved the highest accuracy in all six scenarios. The largest gains occurred under adaptive model poisoning (DBST-FL = 96.32% vs. FedAvg = 80.67% and FLTrust = 82.61%) and semantic backdoor attack (DBST-FL = 97.16% vs. FedAvg = 85.80% and FLTrust = 89.15%), demonstrating that the semantic trust layer detects functional harm that gradient-space methods cannot.

On UNSW-NB15, DBST-FL achieves an accuracy of 0.9799 and an F1 score of 0.9812, remaining competitive with the strongest robust aggregation baselines while achieving stronger backdoor suppression and higher robustness than most Byzantine-robust methods. Coordinate-Wise Median (0.9852) and Trimmed Mean (0.9863) achieved higher raw accuracy on UNSW-NB15 under the full attack mix, reflecting their coordinate-wise robustness on this particular dataset. However, DBST-FL achieved a higher robustness index (0.8838) than FLAME (0.8828), Trimmed Mean (0.8830), Coordinate-Wise Median (0.8811), and all Byzantine-robust baselines except Weighted FedAvg (0.9356). The 15.6 percentage-point gap under adaptive model poisoning compared with Adaptive FedAvg is attributable to DBST-FL’s semantic trust layer detecting functionally harmful updates that preserve gradient-space properties. Figure 6 provides a visual summary across attack types.

### 5.3. Backdoor Defence Performance

Table 6 reports the clean accuracy and attack success rate under trigger-column and semantic backdoor attacks.

**Figure 7:**
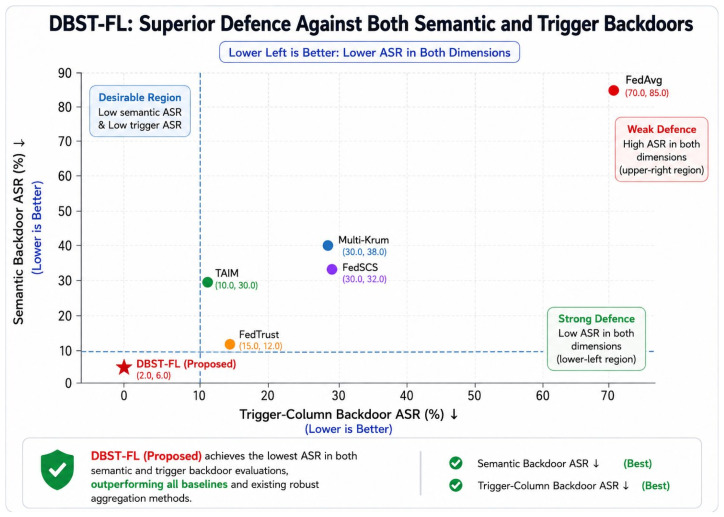
Joint attack success rate (ASR) scatter plot under trigger-column backdoor (x axis) and semantic backdoor (y axis). Dashed lines mark the 10% ASR threshold in both dimensions; the lower-left quadrant is the strong defence region. DBST-FL (red star, lower-left) achieved (2.0%,6.0%)—the lowest ASR in both dimensions simultaneously—well inside the strong defence region. All baselines fell outside: FederatedTrust (15.0%,12.0%), TAIM (10.0%,30.0%), FedSCS (30.0%,32.0%), Multi-Krum (30.0%,38.0%), and FedAvg (70.0%,85.0%).

DBST-FL achieved an ASR of 2.00% under trigger-column and 6.00% under semantic backdoor (Table 6; Figure 7), substantially below FederatedTrust (15.0% and 12.0%, respectively) and TAIM (10.0% and 30.0%, respectively).

The ASR values were obtained as follows. For each method, 500 trigger-activated test samples were presented to the global model at the end of training, and the ASR was computed as the fraction successfully redirected to the attacker target class, averaged over five independent runs with fixed seeds. The trigger-column backdoor used a fixed-column feature pattern; the semantic backdoor used feature-space proximity to an attacker-defined cluster centroid without explicit pixel-level triggers. The clean accuracy (CA) column in Table 6 measures the accuracy on the held-out benign test set under the same five-run protocol. The gap between DBST-FL and FederatedTrust directly quantifies the marginal contribution of the semantic trust layer: 13.0 percentage points under trigger-column backdoor and 6.0 points under semantic backdoor. Figure 7 presents this as a joint ASR scatter plot.

### 5.4. Ablation Study

Table 7 isolates the contribution of each trust component and the fusion strategy.

Both trust layers contributed complementary protection: behavioural trust alone (resilience of 0.5794) suppressed gradient anomalies but missed stealthy backdoors; semantic trust alone (resilience of 0.6169) detected functional harm but was weaker against aggressive poisoning. The key finding is the additive-to-multiplicative gap: identical trust components, different fusion rule. Multiplicative fusion reduced the poisoned weight fraction from 0.1724 to 0.1394 (a 19.1% reduction) and increased resilience from 0.8276 to 0.8606. This improvement is attributable entirely to the non-compensatory gating property (Proposition 1).

**Figure 8:**
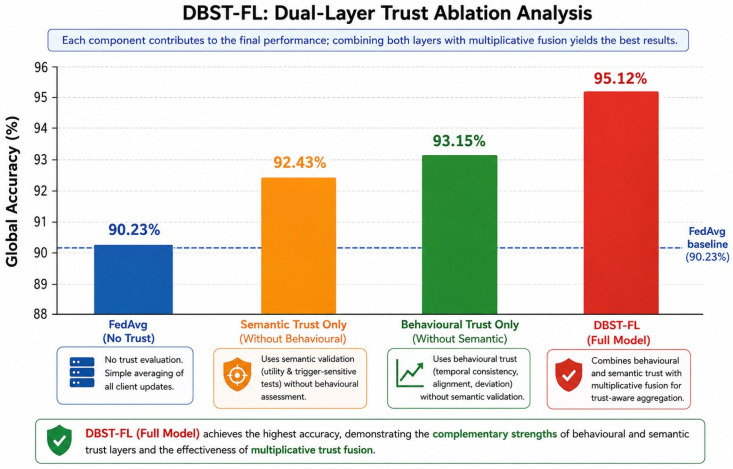
DBST-FL dynamic behavioural and semantic trust ablation analysis. Global accuracy for four configurations: FedAvg baseline (90.23%, no trust), semantic trust only (92.43%, without behavioural assessment), behavioural trust only (93.15%, without semantic validation), and the full DBST-FL model with multiplicative fusion (95.12%). The dashed line marks the FedAvg baseline (90.23%). Each trust layer contributed independently: semantic trust added 2.20 pp over FedAvg; behavioural trust added 2.92 pp; and combining both with multiplicative fusion yielded 4.89 pp improvement. The full model achieved the highest accuracy, confirming the complementary nature of the two trust dimensions.

We note for the metrics that Table 7 reports the *clean accuracy* (accuracy on uncorrupted validation data, 0.9937 for the full DBST-FL configuration) under the semantic-backdoor ablation protocol, whereas Figure 8 reports the *global accuracy* (test accuracy on the held-out global test set averaged across the same four configurations, 95.12% for the full model). These are distinct evaluation protocols measuring different quantities—validation-set utility versus global test-set accuracy—rather than conflicting measurements of the same metric. Figure 8 visualises the per-configuration global test accuracy results.

### 5.5. Performance Under Colluding Adversaries

Table 8 reports DBST-FL’s performance under two coordinated attack scenarios.

DBST-FL retained an accuracy of 0.9443 under continuous collusion and 0.9798 under alternating collusion. However, coordinated adversaries retained 45.7–48.6% of the total aggregation weight, a transparent limitation discussed in Section 6. The higher accuracy under alternating collusion reflects EWMA smoothing; adversaries accumulate positive trust during benign rounds, partially offsetting suppression during attack rounds.

### 5.6. Extended Baseline Comparison

Across 36 experimental conditions (six attacks × three architectures × two datasets), DBST-FL achieved a mean global accuracy of 97.92% and a resilience score of 96.91%, outperforming all evaluated baselines. DBST-FL achieved competitive or superior accuracy compared with FederatedTrust [18], TAIM [25], BC + FL + Trust [44], and FedSCS [24]. Communication overhead averaged 20.50 MB/round, 26.73% below that of TAIM (27.98 MB/round) and 10.81% above that of FederatedTrust (18.50 MB/round). DBST-FL therefore occupies a favourable security-efficiency position: substantially stronger backdoor resistance than FederatedTrust at modest additional overhead and much lower overhead than TAIM with superior or comparable accuracy.

## 6. Limitations

### 6.1. Server-Side Validation Data and Distribution Assumptions

Semantic trust requires a representative clean validation set $D_{\mathrm{val}}$ and trigger-sensitive set $D_{\mathrm{trig}}$. Constructing a representative $D_{\mathrm{val}}$ may be challenging in highly heterogeneous IIoT environments where the server has limited prior knowledge of local data distributions. The trigger-sensitive set improves detection against trigger-like semantic deviations without requiring exact trigger templates but may produce weak initial signals for genuinely novel trigger structures; accumulated evidence across rounds eventually degrades trust for such triggers, but detection may be delayed.

Additional distribution assumptions affect robustness. If $D_{\mathrm{val}}$ is scarce, imbalanced, or drawn from a distribution shifted relative to the client data, then the benign utility score $U_{i}^{t}$ may be a noisy estimator of true client quality. Similarly, if $D_{\mathrm{trig}}$ synthetic perturbations do not cover the specific trigger families used by attackers—such as fully distributed triggers, clean-label backdoors, or attacks optimised to mirror clean-model activations—then the adversarial robustness score $P_{i}^{t}$ may fail to discriminate them. Future work should evaluate DBST-FL under $D_{\mathrm{val}}$ scarcity, missing attack classes, noisy labels, and out-of-domain validation distributions.

### 6.2. Adaptive and White Box Adversaries

The current evaluation considers poisoning and backdoor attacks that are not optimised against the specific trust-scoring functions of DBST-FL. A capable white box adversary with knowledge of both ${\bar{G}}_{i}^{t}$, $H_{i}^{t}$, $D_{i}^{t}$, and $P_{i}^{t}$ could jointly optimise updates to satisfy all four scoring criteria while retaining backdoor functionality. This includes attacks that approximate the $D_{\mathrm{trig}}$ distribution to suppress excess activation, intermittent attacks that exploit EWMA smoothing by appearing benign for several rounds before activating, and coordinated attacks that distribute malicious effects across many clients to remain individually below the isolation threshold. Evaluating DBST-FL against such adaptive attacks is an important direction for future work; preliminary evidence from the collusion experiments (Section 5.5) suggests that even coordinated clients face bounded aggregation weight, which limits but does not eliminate this threat.

### 6.3. Coalition Influence

The dynamic behavioural and semantic trust mechanism evaluates each client independently and does not model inter-client coordination. A coalition distributing malicious contribution across many individually plausible clients may collectively exceed the suppression threshold. As demonstrated in Section 5.5, adversarial coalitions retained 45.7–48.6% of the aggregation weight under the evaluated stress tests. Extending DBST-FL with coalition-aware trust modelling—through graph-based anomaly detection over client interaction patterns or differential discounting for clients with correlated updates—is a priority for future work [13].

### 6.4. Semantic Evaluation Overhead

Semantic trust requires per-round forward passes over validation datasets for each client. While the total complexity is $O(N|D_{\mathrm{val}}|)$ and linear in *N*, the absolute cost depends on the model size and validation set cardinality. In environments with strict latency budgets, adaptive evaluation scheduling may be necessary. Such optimisations were not evaluated in this work.

### 6.5. Deployment Validation

All experiments were conducted in a simulated federated setting on public benchmark datasets. Validation on real industrial edge hardware with authentic traffic, genuine device heterogeneity, and network latency variability is required for deployment readiness. The honest-but-curious server assumption may be too optimistic for some deployment contexts, motivating extension toward privacy-preserving semantic evaluation using homomorphic encryption [45] or secure multi-party computation as future work.

## 7. Conclusions

Federated learning has emerged as a promising paradigm for Industrial Internet of Things (IIoT) environments by enabling collaborative model training without centralising sensitive data. However, the aggregation process remains susceptible to sophisticated poisoning and backdoor attacks, particularly when adversarial updates exhibit characteristics similar to legitimate client contributions. Addressing this challenge requires trust assessment mechanisms that extend beyond conventional gradient-based or reputation-based evaluation approaches.

This paper introduced DBST-FL, a dynamic behavioural and semantic trust framework for robust federated learning in Industrial IoT environments. The proposed approach combines behavioural trust assessment, derived from gradient alignment, historical consistency, and collective deviation analysis, with semantic trust assessment based on model utility and trigger-sensitive validation. A non-compensatory multiplicative fusion mechanism was developed to integrate both trust dimensions, ensuring that strong performance in one trust domain cannot compensate for weaknesses in another. The resulting trust score was incorporated into a trust-aware aggregation strategy that dynamically reduces the influence of potentially malicious participants during model aggregation.

Comprehensive experiments conducted on the Edge-IIoTset and UNSW-NB15 datasets under multiple poisoning and backdoor attack scenarios demonstrated the effectiveness of the proposed framework. The results showed that integrating behavioural and semantic trust provides stronger resilience against adversarial manipulation than existing trust-based aggregation approaches while maintaining high model utility and communication efficiency. Furthermore, ablation studies confirmed the importance of dynamic behavioural–semantic trust evaluation and validated the effectiveness of multiplicative trust fusion in preventing the propagation of malicious updates.

In the proposed method, clients begin each round with a neutral trust history and accumulate evidence across rounds through the EWMA-smoothed historical consistency score. Malicious clients are not immediately excluded; instead, their trust scores decline progressively as behavioural anomalies accumulate, and they are suppressed or isolated once their joint trust score $T_{i}^{t}=B_{i}^{t}\times S_{i}^{t}$ falls below the threshold τ. Benign clients whose updates deviate temporarily (due to non-IID data heterogeneity) are down-weighted rather than excluded, preserving their participation and protecting against false-positive isolation. This gradual, evidence-based suppression mechanism is what distinguishes DBST-FL from hard-threshold defences that risk excluding legitimate participants under heterogeneous data.

The findings of this study demonstrate that robust federated learning can benefit significantly from combining complementary trust perspectives rather than relying on a single assessment dimension. By jointly evaluating behavioural characteristics and semantic outcomes, the proposed framework offers a practical and scalable solution for securing collaborative learning in large-scale Industrial IoT deployments.

Future work will investigate adaptive trust mechanisms capable of evolving in response to changing adversarial strategies, integration with asynchronous and cross-silo federated learning architectures, and evaluation across larger real-world Industrial IoT deployments. Additional research will also explore the incorporation of explainable trust metrics and foundation model-assisted anomaly analysis to further enhance transparency and robustness in trust-aware federated learning systems.

## Figures and Tables

**Table 2:** Computational complexity comparison. DBST-FL achieved linear scaling with the number of clients *N*. Here, *d* = model dimensionality; $|D_{\mathrm{val}}|$ = validation set cardinality; *f* = adversarial client count.

Method	Per-Round	Pairwise?
Multi-Krum [15]	$O(N^{2}d)$	Yes
Bulyan	$O(N^{2}d)$	Yes
Coord. median [16]	O(Nd)	No
FLAME [22]	O(NdlogN)	No
FLTrust [17]	O(Nd)	No
**DBST-FL (this work)**	$O(N(d\;+\;|D_{\mathrm{val}}\left|)\right)$	**No**

**Table 3:** Model architecture details for the three evaluated architectures. All models used cross-entropy loss and the Adam optimiser ($\eta =10^{-3}$). Parameter counts are approximate.

Model	Layers	Drop	Params
ANN	Dense(128, ReLU), Dense(64, ReLU), Dense(*C*, Softmax)	0.2	∼17K
1D-CNN	Conv1D(64,3,ReLU), MaxPool, Conv1D(32,3,ReLU), Flatten, Dense(*C*, Softmax)	0.2	∼25K
LSTM	LSTM(64), Dense(32, ReLU), Dense(*C*, Softmax)	0.2	∼32K

Note: *C* = number of output classes (15 for Edge-IIoTset, 10 for UNSW-NB15). Input shape: tabular feature vector for ANN; feature sequence for 1D-CNN; temporal sequence for LSTM.

**Table 4:** Baseline implementation status and key hyperparameters. ✔ = implemented in unified simulation; ∘ = qualitative or complexity comparison only.

Method	Impl.	Key Hyperparameters
FedAvg	✔	default: lr = 0.001, *E* = 5, *B* = 64
Weighted or Adaptive FedAvg	✔	weights ∝ dataset size; adaptive lr
Krum or Multi-Krum	✔	*f* = 9 Byzantine; multi *m* = 15
Coord. Median or Trimmed Mean	✔	trim ratio = 0.10
FLAME	✔	noise scale σ = 0.01; cluster threshold 0.5
Bulyan	∘	excluded: $O(N^{2}d)$ cost; no IIoT benchmark
FLTrust	✔	server dataset 50 samples; cosine threshold = 0
FederatedTrust	✔	all six trust dimensions; default weights
FedSCS	✔	stable cosine threshold 0.9
TAIM	✔	Stackelberg game; soft filter = 0.5
OptiGradTrust	✔	RL trust; window *K* = 5
BC + FL + Trust	✔	blockchain trust factor; default config
**DBST-FL**	✔	τ = 0.8; *K* = 5; γ = 0.7; $\beta_{1}=\beta_{2}=0.5$

Bold row indicates the proposed method (DBST-FL).

**Table 5:** Mean performance across six adversarial attack scenarios (ANN, 30 clients, 9 adversaries). Acc. = mean adversarial accuracy; F1 = adversarial F1 score; RI = robustness index = worst-case accuracy retention $A_{\mathrm{worst}}/A_{\mathrm{clean}}$, where $A_{\mathrm{worst}}$ is the lowest per-attack accuracy across the six adversarial conditions and $A_{\mathrm{clean}}$ is the no-attack baseline (both measured independently; RI is not derivable from the mean Acc column); SD = standard deviation of Acc. across five independent runs with fixed seeds (1–5). F1 and RI exhibited comparable run-to-run variability (SD≤0.005) and were omitted for table compactness. **Bold** = best result per column (Trimmed Mean achieved the highest Acc and F1 on UNSW-NB15).

Aggregation Method	Edge-IIoTset			UNSW-NB15		
	Acc. (±SD)	F1	RI	Acc. (±SD)	F1	RI
FedAvg [43]	0.9207 ± 0.0041	0.9237	0.7815	0.9739 ± 0.0033	0.9759	0.9245
Weighted FedAvg	0.9197 ± 0.0038	0.9225	0.7795	0.9781 ± 0.0029	0.9797	0.9356
Adaptive FedAvg	0.8472 ± 0.0079	0.8695	0.7324	0.9188 ± 0.0064	0.9269	0.8112
Krum [15]	0.8799 ± 0.0052	0.8854	0.9055	0.9267 ± 0.0047	0.9294	0.8804
Multi-Krum [15]	0.9132 ± 0.0044	0.9161	0.8999	0.9692 ± 0.0036	0.9711	0.8812
Coord. Median [16]	0.9183 ± 0.0040	0.9211	0.9006	0.9852	0.9863	0.8811
Trimmed Mean [16]	0.9212 ± 0.0042	0.9242	0.9006	**0.9863** ± **0.0021**	**0.9873**	0.8830
FLAME [22]	0.9142 ± 0.0046	0.9171	0.9001	0.9601 ± 0.0039	0.9628	0.8828
**DBST-FL (this work)**	**0.9475** ± **0.0019**	**0.9505**	**0.9181**	0.9799 ± 0.0024	0.9812	**0.8838**

Note: Results represent means across label flipping, clean-label poisoning, adaptive model poisoning, explicit overwriting, trigger-column backdoor, and semantic backdoor. Results are from five independent runs. All baseline methods were reimplemented within a unified experimental framework and evaluated using identical datasets, model architectures, client configurations, attack scenarios, and training hyperparameters. Method-specific aggregation parameters were configured according to the recommendations provided in the respective original publications.

**Table 6:** Backdoor defence performance (ANN, 30 clients, 9 adversaries, Edge-IIoTset). CA = clean accuracy; ASR = attack success rate (**↓ lower is better**). DBST-FL achieved the lowest ASR under both attack types while maintaining the highest clean accuracy.

Method	Trigger-Column		Semantic	
	CA (%)	ASR (%) ↓	CA (%)	ASR (%) ↓
FedAvg [43]	94.80	70.00	93.20	85.00
Multi-Krum [15]	95.20	30.00	95.60	38.00
FederatedTrust [18]	96.80	15.00	94.80	12.00
FedSCS [24]	97.70	30.00	95.90	32.00
TAIM [25]	96.60	10.00	95.70	30.00
**DBST-FL (this work)**	**98.20**	**2.00**	**99.50**	**6.00**

**Table 7:** Ablation study of trust components and fusion strategy under semantic backdoor attack (ANN, 30 clients, 9 adversaries, Edge-IIoTset). All configurations used the same ZTA authentication gate. Pois. Wt. Frac. = poisoned weight fraction (**↓ lower preferred**); resilience (**↑ higher preferred**).

Trust Configuration	Clean Acc.	Triggered Acc.	Rob. Index	Pois. Wt. Frac. ↓	Resilience ↑
Behavioural trust only	0.9414	0.8525	0.9056	0.2275	0.5794
Semantic trust only	0.9644	0.8708	0.9030	0.1774	0.6169
Additive fusion (λ=0.5)	0.9864	0.8888	0.9011	0.1724	0.8276
**DBST-FL (multiplicative)**	**0.9937**	**0.8948**	**0.9005**	**0.1394**	**0.8606**

Note: Poisoned weight fraction = total normalised aggregation weight assigned to adversarial clients. **Bold**: best result per metric.

**Table 8:** DBST-FL performance under coordinated colluding-adversary stress tests (ANN, Edge-IIoTset, 30 clients, 9 adversaries). Coalition Weight = total normalised weight assigned to adversarial clients (**lower preferred**). Active Weight = weight assigned to currently attacking adversaries. Coalition Resilience =1− Coalition Weight (**higher preferred**).

Collusion Scenario	Acc.	F1	Rob. Index	Coalition Wt.	Active Wt.	Coalition Res.
Continuous adaptive model poisoning	0.9443	0.9555	0.9198	0.4855	0.4855	0.5145
Alternating collusion	0.9798	0.9833	0.9136	0.4566	0.2255	0.5434

Note: Coalition resilience =1− coalition weight.

## Data Availability

The Edge-IIoTset dataset used in this study is publicly available from the original publication [41]. The UNSW-NB15 dataset is publicly available from the original publication [42]. No new data were created in this study.

## Abbreviations

The following notations and abbreviations are used in this manuscript:

$\theta_{i}^{t}$	Local model update of client *i* at round *t*
$\theta_{\mathrm{global}}^{t}$	Global model at round *t*
$B_{i}^{t}$	Behavioural trust score of client *i* at round *t*
$S_{i}^{t}$	Semantic trust score of client *i* at round *t*
$T_{i}^{t}$	Joint (fused) trust score of client *i* at round *t*
$A_{i}^{t}$	Normalised aggregation weight of client *i* at round *t*
$D_{\mathrm{val}}$	Server-side clean validation dataset
$D_{\mathrm{trig}}$	Server-side trigger-sensitive evaluation dataset
